# Nutritional Counseling in Children with Growth Hormone Deficiency Treated with Recombinant Human Growth Hormone: Analysis of Growth Response Parameters

**DOI:** 10.3390/biomedicines13092165

**Published:** 2025-09-05

**Authors:** Joanna Budzulak, Katarzyna Anna Majewska, Andrzej Kędzia

**Affiliations:** Department of Pediatric Diabetes, Auxology and Obesity, Poznan University of Medical Sciences, 60-572 Poznan, Poland; j.budzulak@gmail.com (J.B.); akedzia@ump.edu.pl (A.K.)

**Keywords:** growth hormone deficiency, nutrition, children, growth hormone treatment

## Abstract

**Background and Objectives:** Growth failure is the main symptom of growth hormone deficiency (GHD) in children. The standard treatment for GHD is the administration of recombinant human growth hormone (rhGH). However, this therapy seems to be less effective in undernourished children. Our study aimed to investigate if professional dietary counseling could affect the parameters of growth response in GHD children undergoing rhGH treatment. **Methods:** The study group involved 29 prepubertal children with GHD (20 boys and 9 girls) aged 8.7 ± 2.50, who were subject to a year of individualized nutritional counseling from a dietician, which included three sessions in 6-month intervals, starting at the beginning of rhGH treatment. The control group consisted of 47 children with GHD (33 boys and 14 girls) aged 8.8 ± 1.73 who were not under the care of the dietician during their rhGH treatment. Parameters of growth response, including the variation of height standard deviation score (ΔhSDS) and height velocity (HV), were compared between the groups during the first two years of rhGH therapy. **Results:** The mean HV and ΔhSDS were slightly higher in the study group than in the control group during both the first and the second year of rhGH therapy, however, those differences did not reach a statistical significance. BMI z-score was stable throughout the observation period. **Conclusions:** One-year of dietary counseling, starting at the beginning of rhGH treatment, and conducted in 6-month intervals did not significantly improve the parameters of growth response in prepubertal GHD children during the first two years of the therapy.

## 1. Introduction

A child’s growth potential primarily depends on genetics; however, the level of its attainment is modulated by endocrine and environmental factors [1,2]. Linear growth is regulated by two key growth-stimulating hormones: growth hormone (GH) and insulin-like growth factor type 1 (IGF-I). GH induces growth directly, acting on tissues through GH receptors, and indirectly, via stimulation of IGF-1 production in the liver [3,4].

Growth hormone deficiency (GHD) is an endocrine disorder with insufficient GH secretion. Although it is a rare disease, it is one of the most important medical causes of short stature among children. There are no precise epidemiological data on the incidence of GHD in Poland. However, the overall estimated prevalence ranges from 1 in 4000 to even 1 in 20,000 [5,6,7,8]. Proper diagnosis is crucial, as the replacement therapy with recombinant human growth hormone (rhGH) is a standard and effective treatment method for GHD [9,10]. However, it is a matter of concern that not all pediatric patients respond sufficiently to this treatment.

Nutrition and nutritional status are both known to influence the GH/IGF-1 axis [1,11,12]. Inadequate energy and protein intake and diet deficient in vitamins and minerals might lower IGF-1 levels [11,13]. Insufficient food intake induces GH peripheral resistance, which results in decreased IGF-1 concentrations. This resistance is also likely to develop against the administered rhGH, leading to unsatisfactory therapy results in undernourished GHD children [13]. Several studies have proved a significant positive correlation between baseline body mass index (BMI) z-score values and height increments during the rhGH treatment in pediatric patients [14,15,16].

Excessive body weight is probably associated with more favorable therapy results [17,18]. It seems that higher BMI values may facilitate adequate energy intake from the diet, which is essential for the effective catch-up growth [15]. However, on the other hand, obesity is thought to be a potential risk factor for developing type 2 diabetes in children treated with rhGH [19].

The aim of this study was to investigate whether professional dietary counseling provided at 6-month intervals during the first year of rhGH treatment affects the parameters of growth response in children with GHD.

## 2. Materials and Methods

### 2.1. Study Design

This study was conducted among prepubertal GHD pediatric patients, treated with rhGH at the Karol Jonscher Memorial Clinical Hospital of the Poznan University of Medical Sciences, in the years 2012–2023. The study group included 29 children (20 boys and 9 girls), with a mean age of 8.7 ± 2.50 (range 5.0–13.0) years. They were recruited from among 36 patients randomly invited to participate in the study after the exclusion of 1 patient diagnosed with celiac disease and 6 patients who manifested signs of puberty. One patient was excluded from further analysis after one year due to treatment discontinuation. Children from the study group received a year of nutritional support from a dietician. The control group consisted of 47 prepubertal children with GHD (33 boys and 14 girls), with a mean age of 8.8 ± 1.73 (range 5.0–13.1) years, who were not counseled by the dietician during the rhGH therapy. Patients from both groups came from the same geographical region of Greater Poland, attended public educational institutions, and used the public healthcare system; hence, their socioeconomic status was similar. All participants had standard physical activity based on classes at school and kindergarten.

For the purpose of the study, data collected from the first two years of rhGH treatment were subject to analysis, as the positive impact of nutritional intervention could occur later than the time of the intervention.

The sample size for this single study was determined regarding representativeness and power, assuming a GHD incidence of 1 per 20,000 [7,8] and 350,000 children aged 5–13 in the region of Greater Poland. Targeting a two-sample, two-sided *t*-test with significance level α = 0.05 and power 1 − β = 0.95, the minimum required size was 13 per group (26 total).

### 2.2. Nutritional Counseling in the Study Group

Patients from the study group were referred to one professional dietician for individualized nutritional counseling. This was based on three sessions, which took place concurrently with routine rhGH treatment follow-up visits: at the beginning of the treatment, then after 6 months and 12 months of rhGH therapy. Information about anthropometric measurements and the patient’s general health status was provided to the dietician by the endocrinologist, who had previously examined the patient on the same day.

During each visit, a dietician assessed the child’s individual energy requirements according to their actual body weight and height measurements. Calorie needs were calculated based on their reported physical activity and the Polish dietary recommendations of the National Food and Nutrition Institute [20,21]. Following this, a dietician conducted a comprehensive nutritional interview with the children and their parents or legal guardians.

Patients were asked to prepare a three-day food diary for each visit, which provided information about meal timings and the amounts of food consumed, specified in both household measurements and grams. At all sessions, children were given questions about eating habits, involving the frequency of daily meals, food preparation methods, the type of fat used in everyday diet, and the habit of sweetening drinks with sugar. At each visit, the children underwent the same dietary survey, which included questions about the frequency of the consumption of different food groups: milk products (or plant-based alternatives), eggs, lean and red meat, fish rich in omega-3 fatty acids, whole grain products, legumes, nuts and seeds, fruit, vegetables as well as highly-processed foods (sweetened beverages, sweets, salty snacks and fast food).

After each visit, all the children and their parents or legal guardians received detailed dietary recommendations, including the advised frequency of meals, daily water consumption, and information about the individual energy requirements. Based on the survey results, a dietician encouraged patients to diversify the types of foods consumed and to limit the consumption of highly processed products. Dietician’s recommendations included following a regular meal pattern, with three to four hours intervals, and reducing the intake of soft drinks and juices, particularly between meals. The intervention aimed to enhance dietary diversity and the consumption of nutrient-dense foods. For this purpose, patients were advised to eat a sufficient amount of products rich in iron (e.g., lean beef, soya, egg yolks, lentils, beans, spinach) and calcium (dairy and calcium-fortified products). Participants were also asked to enrich their diet with vegetables, fruit, nuts, seeds, vegetable oils, and sea fish. All children were given exemplary meal compositions, which provided information about the recommended type and amount of ingredients, helping to meet their energy, macro-, and micronutrient requirements. These recipes considered the patient’s taste preferences.

### 2.3. Inclusion and Exclusion Criteria

All the study participants presented a height SDS (hSDS) below −2 at the start of treatment. Patients were diagnosed with isolated GHD based on the results from two stimulation tests (insulin, clonidine, or glucagon). All of them were prepubertal throughout the observation. Children with intrauterine growth restriction (IUGR) and born small for gestational age (SGA) were excluded from the study. None of them suffered from other chronic diseases, including genetic syndromes, multiple pituitary hormone deficiency, asthma, epilepsy, or malabsorption. All the patients were treated with rhGH - Genotropin^®^ (Pfizer Europe MA EEIG, Bruxelles, Belgium) or Omnitrope^®^ (Sandoz GmbH, Kundl, Austria).

### 2.4. Auxological and Clinical Measurements

All of the children underwent a physical examination by a pediatric endocrinologist during the initial and follow-up visits. Height was measured with a stadiometer with an accuracy of 1 mm. Body weight was assessed to the nearest 0.05 kg. Height SDS (hSDS) was calculated based on the local centile growth charts [22], using the following formula:

$$hSDS=\frac{\left(actual\;height-height\;at\;50th\;centile\right)}{\frac{1}{2}\;\times \left(height\;at\;50th\;centile-height\;at\;3th\;centile\right)}$$

Growth response parameters included: the increment of hSDS (ΔhSDS) and the annual height velocity (HV). HV was calculated as the difference between two height measurements taken at 12-month intervals. Body mass index (BMI; weight (kg)/${\left[height(m)\right]}^{2}$) was converted into z-score values, using Cole’s LMS method [23], according to the Polish growth references [24,25].

$$BMI\;\mathrm{z-}score=\frac{{\left(\frac{BMI}{M}\right)}^{L}-1}{L\times S}$$

### 2.5. Statistical Analysis

The analysis was carried out using IBM SPSS Statistics 28. Statistical significance was determined at a *p*-value < 0.05. In order to establish whether the data were normally distributed, the Shapiro-Wilk test was used. Levene’s test was performed to determine the homogeneity of variance. The non-parametric U-Mann-Whitney test was applied to compare quantitative data between groups, in the case of a lack of normal distribution or homogenous variance, and in other cases, the parametric Student’s *t*-test was used. When comparing three consecutive measurements, in the case of an absence of normal distribution, the Friedman test was chosen. In other cases, the ANOVA test for dependent variables was applied. The chi-square test was chosen to investigate associations between the variables.

## 3. Results

Table 1 and Table 2 present the characteristics of the study and the control group. For both groups, the mean height as well as hSDS improved significantly after 12 and then 24 months of the treatment (*p* < 0.001). The results also indicate that the mean body weight in the two groups increased significantly after one and two years of therapy (*p* < 0.001). However, mean BMI z-score values did not change significantly between the consecutive follow-up visits in both the study group (*p* = 0.965) and the control group (*p* = 0.353).

The analysis also included a comparison of the variables between the two groups. Initially, the proportion of girls and boys was similar in both groups (*p* = 0.908). The evaluation of bone age at the treatment entry presented no significant differences between the study and the control group (7.1 ± 2.9 and 7.0 ± 2.0 years, respectively; *p* = 0.889). Also, there were no statistically significant differences in the mean age (*p* = 0.564), hSDS (*p* = 0.231), as well as BMI z-score values (*p* = 0.390) between the groups. Mean BMI z-score values did not differ significantly at 12-month (*p* = 0.744) and 24-month (*p* = 0.810) follow-up visits (Supplemental Graph S1). Finally, there was no significant difference in mean hSDS after 12 months (*p* = 0.312) and 24 months (*p* = 0.713) of the therapy. Detailed information is presented in Table 1 and Table 2.

In order to investigate whether dietary intervention impacts the results of rhGH treatment, we compared the parameters of growth response (Table 3). During the first year of rhGH therapy, in the study group HV and annual increase in hSDS (ΔhSDS) had slightly higher mean values than in the control group, but these differences did not reach statistical significance. Similar results were recorded in the second year of treatment. Visual data regarding HV trajectory is provided in Supplemental Graph S2. Finally, our study also evaluated the impact of dietary counseling on the nutritional status of participants, expressed as an annual increase in the BMI z-score (ΔBMI z-score). The obtained results indicate no statistically significant differences between the study and the control group in the mean ΔBMI z-score during the first and the second year of the therapy.

## 4. Discussion

According to numerous studies, the function of the growth hormone (GH) and insulin-like growth factor type 1 (IGF-1) axis is clearly affected by nutrients and nutritional status [1,3,12,26]. Particular attention has been paid to the impact of energy and protein intakes. It seems that both calorie and protein deficits might cause a state of GH resistance that has been hypothesized as an adaptive mechanism of preserving energy during undernutrition [13,27]. It is likely that elevated GH levels support preventing hypoglycemia and mobilization of fatty acids from adipose tissue [27,28]. Even though those alterations are more pronounced during severe protein-calorie malnutrition and fasting, they could also develop due to short-term and mild food restrictions [13,26,29]. The state of GH resistance might also explain a poor response to recombinant human growth hormone (rhGH) treatment in undernourished children with GHD [13].

Our previous analysis, including 80 prepubertal GHD children, has shown a positive correlation between the initial mean body mass index (BMI) z-score and height velocity (HV) during the first year of rhGH treatment [15]. Another study, conducted by Majcher et al., has proved that prepubertal GHD children with normal or excessive body weight present significantly better HV during the first year of therapy, compared to underweight participants [18]. Children with lower body weight seem to consume fewer calories and, therefore, do not fully meet the energy requirements needed for accelerated growth, which is observed at the beginning of treatment [15,30].

Obesity is usually associated with reduced GH secretion but probably increased GH sensitivity, and IGF-I bioavailability [13,31,32]. Yang et al. have reported that obese GHD children achieve better rhGH therapy results during the first two years of therapy, compared to non-obese individuals [17]. Better increase in height in obese children might be partly explained by relatively high doses of prescribed rhGH [33,34], as the dose is based on body weight. However, long-term treatment outcomes seem less satisfactory in individuals with excessive body weight [5]. Moreover, obesity probably enhances the risk for developing type 2 diabetes in children treated with rhGH [19]. Therefore, the appropriate management of nutritional status could potentially increase the safety and effectiveness of the treatment.

In this study, we aimed to investigate whether a one-year nutritional support from a dietician, conducted during the first year of rhGH treatment in 6-month intervals, allowed the achievement of more favorable therapy results. The primary response to treatment is thought to be a reliable predictor of its long-term outcome [35]; thus, there is a need to identify factors that could contribute to better height gains during this period. We analyzed the effectiveness of one-year dietary counseling throughout the first two years of rhGH therapy, because the impact of our intervention could be seen later than the time of its duration. However, despite slightly higher mean HV and an increase in height SDS (ΔhSDS) in the study group, observed differences were not statistically significant. 

The results also show no statistically significant differences between the two groups in mean BMI z-score values at the treatment entry and after 12 months and 24 months of the treatment. Moreover, the mean BMI z-score has not changed significantly throughout all visits in both groups. Finally, we found no statistically significant differences in the mean ΔBMI z-score between the groups during the therapy’s first and second years. The support of a dietician did not influence BMI z-score values, and this might partly explain the similar response to rhGH treatment between the study and the control group.

As several studies prove that initial BMI z-score values, evaluated before the therapy beginning, influence growth outcomes in children subsequently treated with rhGH [15,16,17,36], we hypothesize that our intervention might not have been successful because it started at the time of the treatment’s initiation. Perhaps taking earlier action to improve nutrition before this therapy would have brought better results.

In our study, a qualified dietician prepared individual recommendations based on information about actual anthropometric measurements, the patient’s dietary habits, as well as their general health status. In Poland, monitoring rhGH therapy in GHD children does not routinely include screening for specific nutrient deficiencies [37]. We believe identifying nutrient deficiencies in GHD patients could enable more effective dietary intervention, thereby allowing for more specific targeted supplementation.

Our intervention focused on providing general dietary guidance aimed at meeting all nutritional requirements. These recommendations were individualized and took into account the food preferences of each patient. All of the children, along with their parents or legal guardians, were given sample recipes to support adherence to the dietician’s advice. However, dietician care consisted of only three sessions at six-month intervals, which took place on the same day as scheduled medical appointments. According to Lifshitz, pediatric patients might need more frequent follow-up visits to reinforce nutrition knowledge and promote healthy eating behaviors [38]. Therefore, future studies in this area should consider more intensive dietary intervention among GHD children. Furthermore, receiving dietary counseling does not guarantee adherence to the recommendations. It is possible that even with the appropriate nutritional advice, parental compliance may have been insufficient, potentially resulting in an imbalanced diet. According to statistics, fussy eating behaviors might affect up to 22% of children over 2.5 years old [39]. This behavioral trait hinders following dietary advice and might require a more comprehensive approach, including an in-depth evaluation of parent-child feeding interaction [40].

We have identified several strengths and limitations of this study. Among the strengths, the groups were homogenous in terms of gender, metrical and bone age, hSDS, as well as BMI z-score at baseline. These parameters are known to affect the outcomes of rhGH therapy [15,41,42]. Moreover, all participants from both groups remained prepubertal throughout the observation period, thereby minimizing the influence of sex hormones on growth outcomes. The limitation of this study is the relatively small sample size of the intervention group, which may have prevented differences in growth response parameters from reaching statistical significance. However, GHD is a rare condition, and additionally, for the purpose of the study, we have excluded patients with signs of puberty. This significantly reduced the number of participants, but it was essential to conduct a reliable analysis. Another limitation was the matter of compliance—the implementation and adherence to dietary recommendations depended on parents and patients, where verification could be based solely on their declaration and was not possible to be performed objectively. Even though food diaries are commonly used for dietary assessment, they might provide inaccurate data. Methods helping to monitor patients’ compliance with dietary advice, such as compliance scores, still rely on parental engagement.

## 5. Conclusions

Dietary counseling conducted at 6-month intervals in the first year of rhGH treatment was not sufficient to allow for effective, statistically significant improvement of growth parameters in children with GHD. This observation was consistent with a stable nutritional status that did not change despite dietician care. We believe that in future studies, the effect of nutritional intervention could be improved by increasing the frequency of consultations, consistently encouraging parents and children to follow nutritional recommendations, and including education on the importance of nutrition for the proper development of children. Furthermore, patients with GHD could probably benefit more from earlier nutritional intervention implemented prior to the initiation of rhGH therapy.

## Figures and Tables

**Table 1:** The characteristics of the study group at all nutritional follow-up visits.

The Variables	Initial Visit	12-Month Follow-Up Visit	24-Month Follow-Up Visit
Total number of subjects	*n* = 29	*n* = 29	*n* = 28
Gender F/M	9 (31%)/20 (69%)	9 (31%)/20 (69%)	8 (29%)/20 (71%)
Age in years, mean (SD)	8.7 (2.5)	9.7 (2.5)	10.7 (2.5)
Body height in cm, mean (SD)	118.2 (14.0)	127.3 (14.1)	134.9 (14.8)
hSDS, mean (SD)	−3.01 (0.79)	−2.20 (0.60)	−1.77 (0.60)
Body weight, mean (SD)	22.34 (7.72)	26.82 (9.63)	31.07 (11.34)
BMI z-score, mean (SD)	−0.66 (0.68)	−0.63 (0.89)	−0.61 (0.97)

hSDS height standard deviation score; BMI body mass index.

**Table 2:** The characteristics of the control group at all nutritional follow-up visits.

The Variables	Initial Visit	12-Month Follow-Up Visit	24-Month Follow-Up Visit
Total number of subjects	*n* = 47	*n* = 47	*n* = 47
Gender F/M	14 (30%)/33 (70%)	14 (30%)/33 (70%)	14 (30%)/33 (70%)
Age in years, mean (SD)	8.8 (1.73)	9.8 (1.73)	10.8 (1.73)
Body height in cm, mean (SD)	119.1 (8.69)	127.8 (8.67)	135 (9.08)
hSDS, mean (SD)	−2.79 (0.61)	−2.05 (0.65)	−1.71 (0.72)
Body weight, mean (SD)	22.58 (4.87)	26.61 (5.97)	30.83 (7.66)
BMI z-score, mean (SD)	−0.50 (0.86)	−0.56 (0.93)	−0.51 (0.95)

hSDS height standard deviation score; BMI body mass index.

**Table 3:** A comparison of the mean HV, ΔhSDS and ΔBMI z-score during the first two years of rhGH treatment between the study and the control group.

The Period of Treatment	The Parameter	The Mean (SD) for the Study Group	The Mean (SD) for the Control Group	*p* Value
The first year of rhGH treatment (the intervention period)	HV	9.14 (1.68)	8.79 (1.33)	0.432
	ΔhSDS	0.81 (0.42)	0.74 (0.33)	0.438
	ΔBMI z-score	0.03 (0.51)	−0.06 (0.28)	0.560
The second year of rhGH treatment	HV	7.51 (1.59)	7.17 (1.34)	0.366
	ΔhSDS	0.41 (0.28)	0.34 (0.22)	0.217
	ΔBMI z-score	−0.01 (0.36)	0.05 (0.36)	0.669

HV height velocity (cm per year); hSDS height standard deviation score; BMI body mass index. ΔhSDS—calculated as the difference in hSDS values between the end and the beginning of the analyzed period. ΔBMI z-score—calculated as the difference in BMI z-score values between the end and the beginning of the analyzed period.

## Data Availability

Data analyzed during this study are available on a reasonable request.

## Supplementary Materials

The following supporting information can be downloaded at: https://www.mdpi.com/article/10.3390/biomedicines13092165/s1, Graph S1: Line graph presenting mean BMI z-score values noted at initial visit, after 12 and 24 months of rhGH therapy in the study and the control group; Graph S2: Line graph presenting mean height velocity (HV) during the first and the second year of rhGH therapy in the study and the control group.

## Abbreviations

The following abbreviations are used in this manuscript:

GHD	growth hormone deficiency
GH	growth hormone
IGF-1	insulin-like growth factor type 1
rhGH	recombinant human growth hormone
hSDS	height standard deviation score
HV	height velocity
BMI	body mass index
IUGR	intrauterine growth restriction
SGA	small for gestational age

## References

[1] Inzaghi E., Pampanini V., Deodati A., Cianfarani S. (2022). The Effects of Nutrition on Linear Growth. Nutrients.

[2] Majewska K.A., Tchorzewska-Skrobich M., Wais P., Majewski D., Naskręcka M., Kędzia A. (2024). Deficient or Normal Growth Hormone Secretion in Polish Children with Short Stature: Searching for Clinical Differences. Biomedicines.

[3] Blum W.F., Alherbish A., Alsagheir A., El Awwa A., Kaplan W., Koledova E., Savage M.O. (2018). The growth hormone-insulin-like growth factor-I axis in the diagnosis and treatment of growth disorders. Endocr. Connect..

[4] Devesa J., Almengló C., Devesa P. (2016). Multiple Effects of Growth Hormone in the Body: Is it Really the Hormone for Growth?. Clin. Med. Insights Endocrinol. Diabetes.

[5] Maroszczuk T., Kapała J., Sitarz A., Kącka-Stańczak A., Charemska D. (2024). Does excessive body mass affect the rhGH therapy outcomes in GHD children?. Pediatr. Endocrinol. Diabetes Metab..

[6] Stanley T. (2012). Diagnosis of growth hormone deficiency in childhood. Curr. Opin. Endocrinol. Diabetes Obes..

[7] Sharma H., Mathur S.K., Purwar N., Sahlot R., Garg U., Sharma B. (2021). Clinical and biochemical phenotype of Indian children with different types of idiopathic growth hormone deficiency and their association with pituitary height on MRI. Indian. J. Endocr. Metab..

[8] Mameli C., Guadagni L., Orso M., Calcaterra V., Wasniewska M.G., Aversa T., Granato S., Bruschini P., d’Angela D., Spandonaro F. (2024). Epidemiology of growth hormone deficiency in children and adolescents: A systematic review. Endocrine..

[9] Maghnie M., Ranke M.B., Geffner M.E., Vlachopapadopoulou E., Ibáñez L., Carlsson M., Cutfield W., Rooman R., Gomez R., Wajnrajch M.P. (2022). Safety and Efficacy of Pediatric Growth Hormone Therapy: Results From the Full KIGS Cohort. J. Clin. Endocrinol. Metab..

[10] Guzzetti C., Ibba A., Incandela V., Loche S. (2024). GH Therapy in Non-Growth Hormone-Deficient Children. Children.

[11] Budzulak J., Majewska K.A., Kędzia A. (2022). Malnutrition as the cause of growth retardation among children in developed countries. Ann. Agric. Environ. Med..

[12] Caputo M., Pigni S., Agosti E., Daffara T., Ferrero A., Filigheddu N., Prodam F. (2021). Regulation of GH and GH Signaling by Nutrients. Cells.

[13] Hawkes C.P., Grimberg A. (2015). Insulin-Like Growth Factor-I is a Marker for the Nutritional State. Pediatr. Endocrinol. Rev..

[14] Lee P.A., Germak J., Gut R., Khutoryansky N., Ross J. (2011). Identification of factors associated with good response to growth hormone therapy in children with short stature: Results from the ANSWER Program^®^. Int. J. Pediatr. Endocrinol..

[15] Budzulak J., Majewska K.A., Kędzia A. (2024). BMI z-score as a prognostic factor for height velocity in children treated with recombinant human growth hormone due to idiopathic growth hormone deficiency. Endocrine.

[16] Cho W.K., Ahn M.B., Kim E.Y., Cho K.S., Jung M.H., Suh B.K. (2020). Predicting First-Year Growth in Response to Growth Hormone Treatment in Prepubertal Korean Children with Idiopathic Growth Hormone Deficiency: Analysis of Data from the LG Growth Study Database. J. Korean Med. Sci..

[17] Yang A., Cho S.Y., Kwak M.J., Kim S.J., Park S.W., Jin D.-K., Lee J.-E. (2019). Impact of BMI on peak growth hormone responses to provocative tests and therapeutic outcome in children with growth hormone deficiency. Sci. Rep..

[18] Majcher A., Czerwonogrodzka-Senczyna A., Woźniak E., Pyrżak B. (2014). Effect of nutritional status on growth velocity in the first year of growth hormone treatment of children with Growth hormone deficiency. Postępy Nauk. Med..

[19] Cianfarani S. (2021). Safety of Pediatric rhGH Therapy: An Overview and the Need for Long-Term Surveillance. Front. Endocrinol..

[20] Jarosz M. (2017). Nutrition Standards for the Polish Population.

[21] Jarosz M. (2020). Nutrition Standards for the Polish Population.

[22] Palczewska I., Niedzwiedzka Z. (2001). Somatic development indices in children and youth of Warsaw. Med. Wieku Rozw..

[23] Cole T.J. (1990). The LMS method for constructing normalized growth standards. Eur. J. Clin. Nutr..

[24] Kułaga Z., Grajda A., Gurzkowska B., Góźdź M., Wojtyło M., Świąder A., Różdżyńska-Świątkowska A., Litwin M. (2013). Polish 2012 growth references for preschool children. Eur. J. Pediatr..

[25] Kułaga Z., Litwin M., Tkaczyk M., Palczewska I., Zajączkowska M., Zwolińska D., Krynicki T., Wasilewska A., Moczulska A., Morawiec-Knysak A. (2011). Polish 2010 growth references for school-aged children and adolescents. Eur. J. Pediatr..

[26] Ban B., Zhao Q. (2018). Nutritional Regulation of Growth Hormone/Insulin-like Growth Factor-1 Axis. Nutri. Food Sci. Int. J..

[27] Fazeli P.K., Klibanski A. (2014). Determinants of GH resistance in malnutrition. J. Endocrinol..

[28] Fedorczak A., Lewiński A., Stawerska R. (2023). Involvement of Sirtuin 1 in the Growth Hormone/Insulin-like Growth Factor 1 Signal Transduction and Its Impact on Growth Processes in Children. Int. J. Mol. Sci..

[29] Haines M.S. (2023). Endocrine complications of anorexia nervosa. J. Eat. Disord..

[30] Kędzia A., Majewska K.A. (2023). Growth velocity increase in children with Turner syndrome treated with growth hormone following a brief therapy interruption. Pediatr. Pol.-Pol. J. Paediatr..

[31] Lee N.Y., Kim S.E., Kim S., Ahn M.B., Kim S.H., Cho W.K., Cho K.S., Jung M.H., Suh B.-K. (2021). Effect of body mass index on peak growth hormone level after growth hormone stimulation test in children with short stature. Ann. Pediatr. Endocrinol. Metab..

[32] Lewitt M.S., Dent M.S., Hall K. (2014). The Insulin-Like Growth Factor System in Obesity, Insulin Resistance and Type 2 Diabetes Mellitus. J. Clin. Med..

[33] Hawcutt D.B., Bellis J., Price V., Povall A., Newland P., Richardson P., Peak M., Blair J. (2017). Growth hormone prescribing and initial BMI SDS: Increased biochemical adverse effects and costs in obese children without additional gain in height. PLoS ONE.

[34] Yoon J.Y., Cheon C.K., Lee J.H., Kwak M.J., Kim H.-J., Kim Y.J., Lee J.E., Chung W.Y., Kim J., Yoo J.-H. (2022). Response to growth hormone according to provocation test results in idiopathic short stature and idiopathic growth hormone deficiency. Ann. Pediatr. Endocrinol. Metab..

[35] Kapała J.M., Maroszczuk T., Sitarz A., Kącka A., Charemska D. (2024). Primary response in GHD children treatment as a predictor for long-term therapy effectiveness therapy effectiveness. Pediatr. Endocrinol. Diabetes Metab..

[36] Esen I., Demirel F., Tepe D., Kara O., Koc N. (2013). The association between growth response to growth hormone and baseline body composition of children with growth hormone deficiency. Growth Horm. IGF Res..

[37] Zdrowia M. (2025). Obwieszczenie Ministra Zdrowia z dnia 17 czerwca 2025 r. w Sprawie Wykazu Refundowanych Leków, Środków Spożywczych Specjalnego Przeznaczenia Żywieniowego Oraz Wyrobów Medycznych na 1 Lipca 2025 r. Programy Lekowe. Załącznik nr B19: Leczenie Niskorosłych Dzieci z Somatotropinową Niedoczynnością Przysadki (ICD-10 E23). https://www.gov.pl/attachment/8a82fe3b-f245-4810-8085-9444c799e6a4.

[38] Lifshitz F. (2009). Nutrition and growth. J. Clin. Res. Pediatr. Endocrinol..

[39] Smolko N.A., Valiev R.I., Kabdesh I.M., Fayzullina R.A., Mukhamedshina Y.O. (2024). Eating disorder in children: Impact on quality of life, with a spotlight on autism spectrum disorder. Nutr. Res..

[40] Walton K., Kuczynski L., Haycraft E., Breen A., Haines J. (2017). Time to re-think picky eating?: A relational approach to understanding picky eating. Int. J. Behav. Nutr. Phys. Act..

[41] Kędzia A., Majewska K.A., Korcz M. (2020). Do the intervals in growth hormone therapy positively affect the growth velocity?. Pediatr. Endocrinol. Diabetes Metab..

[42] Ross J., Fridman M., Kelepouris N., Murray K., Krone N., Polak M., Rohrer T.R., Pietropoli A., Lawrence N., Backeljauw P. (2023). Factors Associated With Response to Growth Hormone in Pediatric Growth Disorders: Results of a 5-year Registry Analysis. J. Endocr. Soc..

