# Supplementary material for: Nutritional Counseling in Children with Growth Hormone Deficiency Treated with Recombinant Human Growth Hormone: Analysis of Growth Response Parameters
Source: Biomedicines. 2025 Sep 5;13(9):2165. doi: 10.3390/biomedicines13092165 (PMC12467744; doi:10.3390/biomedicines13092165)

Supplemental Graph S1. Line graph presenting mean BMI z-score values noted at initial visit, after 12 and 24 months of rhGH therapy in the study and the control group.

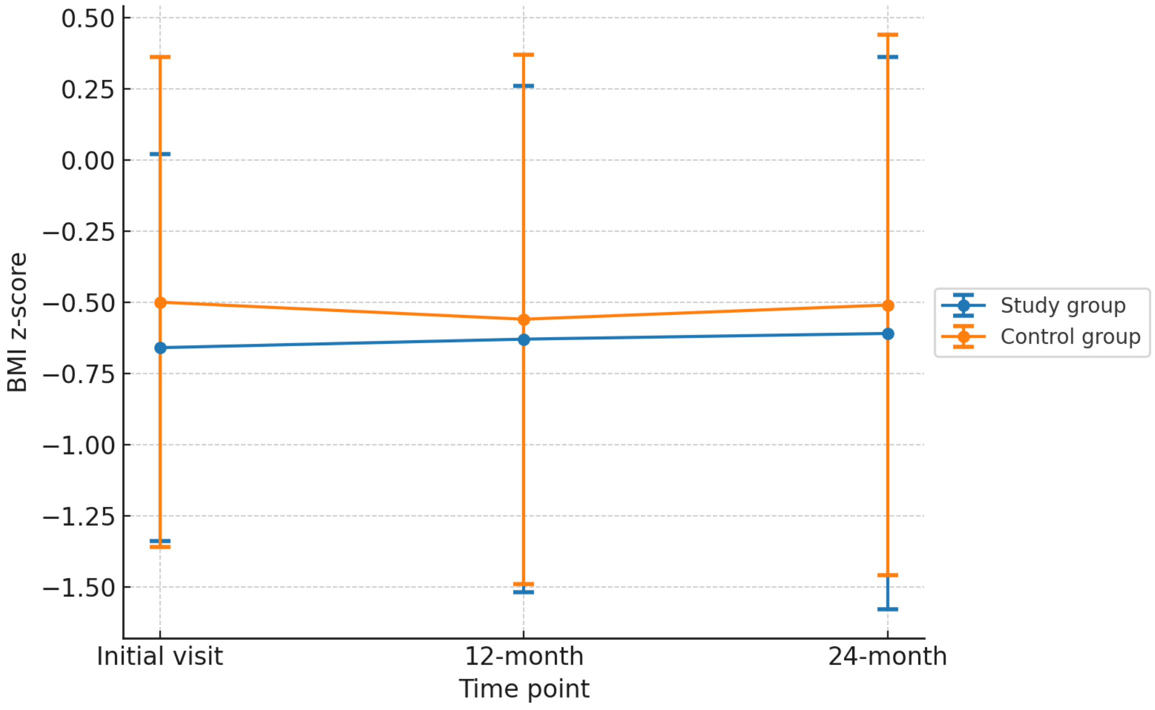

Supplemental Graph S2. Line graph presenting mean height velocity (HV) during the first and the second year of rhGH therapy in the study and the control group.

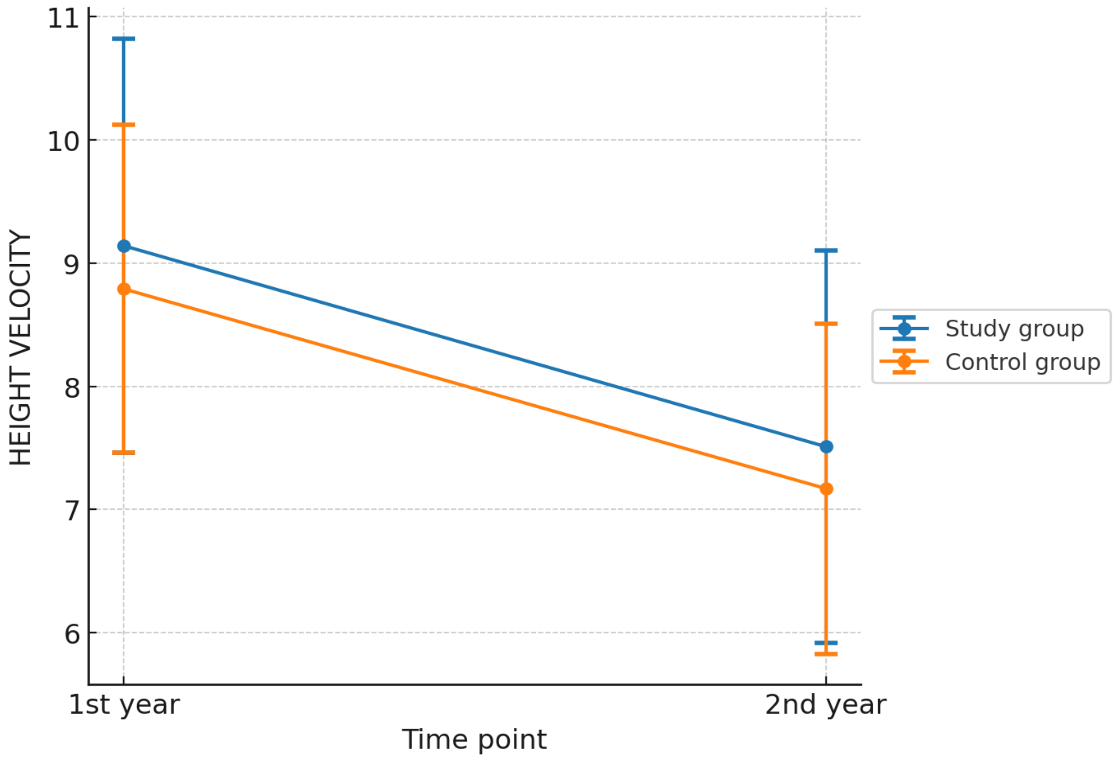

Supplement: Supplementary file 1 [file biomedicines-13-02165-s001.zip › biomedicines-3778037-supplementary.pdf]
